# Gaps, Successes, and Opportunities Related to Social Drivers of Health from the Perspectives of Black Preterm Infant Caregivers: A Qualitative Study

**DOI:** 10.1016/j.jpeds.2025.114598

**Published:** 2025-04-16

**Authors:** Kayla L. Karvonen, Erica Anunwah, Serena Gilmore, Ursula Griffiths-Randolph, Kristine A. Karvonen, Dishon Moore, Kobi Miller, Josten Overall, Lawren Wooten, Patience A. Afulani

**Affiliations:** 1 Department of Pediatrics, University of California San Francisco, San Francisco, CA; 2 Department of Pediatrics, University of California San Diego, San Diego, CA; 3 School of Medicine, University of California San Francisco, San Francisco, CA; 4 Division of Public Health Sciences, Fred Hutchinson Cancer Center, Seattle, WA; 5 Division of Pediatric Hematology/Oncology, Department of Pediatrics, University of Washington School of Medicine, Seattle, WA; 6 Department of General Pediatrics, Denver Health Medical Center, Denver, CO; 7 Department of Obstetrics Gynecology and Reproductive Sciences, University of California San Francisco, San Francisco, CA

## Abstract

**Objective:**

To identify Black preterm infant caregiver experiences and institutional priorities regarding screening and addressing social drivers of health (SDH).

**Study design:**

In the Centering Black Preterm Infant Caregiver Priorities study, Black female researchers conducted semistructured interviews in 2024 with Black caregivers of preterm infants born in the San Francisco Bay Area of California. Transcripts were coded using a book generated from an interview guide, and the resulting data were analyzed using thematic analysis. Themes were generated and refined through discussion.

**Results:**

Twenty sociodemographically diverse caregivers participated. Five themes were identified: (1) financial insecurity and inadequate access to resources for everyday social needs contribute negatively to caregiver and child health and well-being; (2) a trusted provider who takes a personal approach to screening and addressing SDH is needed in medical settings; (3) inequitably distributed, fragmented, and disorganized medical and social support systems in the transition to home period are burdensome and a source of stress; (4) community-based organizations centering Black families holistically address SDH and promote social well-being and connectedness; and (5) state and federal legislation, policies, and programs are critical opportunities to address SDH.

**Conclusions:**

SDH are a significant source of stress for caregivers after preterm birth, and there are opportunities across state and federal legislative policies, community-based organizations, medical systems, and connections across the systems to address them.

Preterm birth and its consequences, including comorbidities, neurodevelopmental impairment, death, and high health care utilization, are racially patterned.^1–6^ Race is a social construct and racially marginalized communities, like Black families, are at higher risk of each compared with other racialized groups due to the downstream effects of racism.^7,8^ Social drivers of health (SDH) are mediators between structural racism and adverse health outcomes and can be acted upon to improve health and reduce health care utilization in general pediatric populations.^9^ SDH are multifaceted and examples include access to housing, food, utilities, transportation, educational and employment opportunities, and health care.^10^ Compared with the general pediatric population, Black families of preterm infants are at greater risk for unique challenges, such as exposure to structural and interpersonal racism. As a result, they face a higher burden of social needs, complex medical conditions, and high health care utilization; thus are poised to benefit from comprehensive, culturally competent SDH interventions.^1,3,11^

Addressing SDH has the potential to improve neurodevelopmental outcomes for preterm infants. For example, facilitating access to health care services like early intervention programs has the potential to improve cognitive and motor development.^12^ Randomized controlled trials have demonstrated systematic SDH screening can be successfully implemented^9^ and interventions on SDH can be efficacious in improving rates of immunizations, preventive care visits, parent reports of overall child health, and health care utilization.^11,13–15^

Although there is potential for SDH screening and interventions to improve child health outcomes, particularly for Black communities who are at higher risk for experiencing SDH, it is not yet known how these strategies may be utilized or enhanced to impact Black preterm infant health. In recent studies, Black caregivers with preterm infants have provided insights on the hospitalization period,^16^ racism,^17–19^ stress,^20^ and mental health.^21^ By revealing the lived experiences and perspectives of marginalized communities, we can expand our knowledge of screening and addressing SDH to provide meaningful data that are critical for the remediation of racial inequities and the advancement of health for Black preterm infants.

In the Centering Black Preterm Infant Caregiver Priorities (CENTER and CARE) Study, we interviewed Black preterm infant caregivers to identify priorities to inform future interventions that improve Black preterm infant health.^22^ In this first analysis, we sought to understand caregiver experiences with care as usual and priorities regarding screening and addressing SDH. A forthcoming analysis illuminates caregiver perspectives on internal and interpersonal mechanisms of long-term dyadic thriving.

## Methods

### Settings and Participants

This study was conducted in the San Francisco Bay Area of California, where 11%−15% of Black birthing people give birth to preterm infants, amongst the highest rates compared with other racialized groups.^23^ There is no standardization to SDH screening and intervention across local neonatal intensive care units (NICUs) or outpatient clinics. We used convenience and purposive sampling to select participants. First participants were conveniently recruited remotely, in-person, and by phone, via social media, flyers in outpatient pediatrics medical settings throughout the San Francisco Bay Area, California, and direct outreach to participants identified as eligible from electronic medical records. Potentially eligible participants were purposively sampled by screening and inclusion if they met the eligibility criteria, which included caregivers who identify as Black/African American and who have had preterm infants (<35 weeks of gestational age) in any NICU in the Bay area. Caregivers who identified with more than 1 race, in addition to Black/African American, were included. The exclusion criteria initially included caregivers whose preterm infants were >10 years old at time of study, a timeframe chosen by the research team to limit recall bias. However, this timeframe was subsequently expanded during recruitment to include children up to 12 years of age at the time of the study to increase the pool of eligible respondents.

### Study Procedures

Three Black female identifying researchers (E.A., S.G., J.O.) in various stages of medical training conducted virtual interviews with caregivers between January and September 2024, using a semistructured interview guide (Supplementary Appendix; available at www.jpeds.com). The interview guide was developed by the study team, including a Black female identifying community advisor, and practiced between researchers. The socioecological model, which proposes that health and development is determined by multiple levels of social and environmental influences,^24^ was used to guide the development of the interview guide. The guide was developed to explore (1) factors across socioecological levels that were barriers or facilitators to their child’s thriving and (2) experiences with SDH screening and interventions (Supplementary Appendix; available at www.jpeds.com). An initial deductive codebook was created from the interview guide and inductive codes were added and refined during transcript review. In addition, critical race theory praxis was also utilized to center this racially marginalized group.^25^ Critical race theory also guided the composition of the research team, which composed of Black women who both share and have unique lived experiences in regard to experiences of racism, SDH, caregiving, and prematurity. The researchers conducting interviews had varied prior research experience, but all participated in a training on qualitative interviewing and data analysis led by an experienced qualitative researcher. Racial concordance between researchers and participants maximized psychological safety for participants to share, and virtual interviews were utilized for maximize convenience. A demographic sheet was administered before the interview. The research team conducted virtual interviews using Zoom with mandatory video “on camera” recording that were planned for 1 hour and lasted 28–96 minutes (mean 52, SD 16.3). Interviewers took field notes during the sessions and did not have prior relationships with participants. Sessions were audio and video recorded and transcribed by a non-artificial intelligence based reputable transcription company. Participants received remuneration of $100 for their time. The University of California San Francisco Institutional Review Board reviewed this study and was determined to be exempt (IRB # 23–39452). All participants provided verbal informed consent. The study was performed in accordance with the Declaration of Helsinki.

### Data Analysis

Transcripts were uploaded to Dedoose for data management and data were analyzed using thematic analysis. Three Black female researchers (L.W., E.A., J.O.) functioned as 3 independent coders. A preliminary deductive codebook was developed with anticipated relevant concepts. After coding small 3–5 batches of transcripts, the research team met to review excerpts and their codes, and to reach a consensus on any disagreements. The analysis team then developed a final codebook that included both initial deductive and additional inductive codes, which were used to code the rest of the transcripts. Following coding, themes were developed and refined through discussion with the analysis team over several sessions with consensus reached regarding themes and further discussion with the entire study team. Data for each theme were then summarized with representative quotes. Thematic saturation was reached by interview 15, in that no new themes were identified. After the analysis, the manuscript was reviewed, and participant feedback was received and incorporated.

### Reflexivity Statement

Our team consisted of 10 Black women with undergraduate and postgraduate level training in medicine, nursing, public health, and psychology. This racial concordance and multidisciplinary nature strengthened our study team as our lived experiences informed the development and implementation of the study and allowed our participants to engage with the study team more comfortably. All authors contributed to the data analysis or interpretation and manuscript writing. We recognize that these identities shape the ways in which we view the world, hence how we interpret information. Thus, we ensured our interpretation was grounded in the data and shared the manuscript with participants for member checking to increase validity.

## Results

Twenty Black caregivers with preterm infants were interviewed for this study (see Table I for participant demographics). Most caregivers identified as female and were born in the US (n = 18, 90%). Caregivers identified across the Black diaspora: African American/Black (n = 15, 75%), Black African (n = 2, 10%), African American/Native (n = 1, 5%), Afro Latinx (n = 1, 5%), and Afro Caribbean (n = 1, 5%). Caregiver household income was categorized as <$10 000/year (n = 6), $10 000–49 000/year (n = 4), $50 000–100 000k/year (n = 4), and >$100 000/year (n = 5). Caregivers had similar rates of private (n = 9) to public insurance (n = 10), and 1 caregiver did not have insurance. Child gestational age at birth ranged from 23–35 weeks (mean 27.5, SD 5.8) and child age at study was 0–12 years (mean 6.0, SD 4.1). Eight hospitals in the Bay Area were represented.

We identified 5 themes from the data spanning several socioecological levels (Table II): at the individual level, (1) financial insecurity and inadequate access to resources for everyday social needs negatively contribute to caregiver and child health and well-being; at the interpersonal level, (2) a trusted provider who takes a personal approach to screening and addressing SDH is needed in medical settings; at the institutional level, (3) Inequitably distributed, fragmented, and disorganized medical and social support systems in the transition to home period is burdensome and a source of stress; at the community level, (4) community-based organizations centering Black families holistically address SDH and promote social well-being and connectedness; at the policy level, (5) state and federal legislation, policies, and programs are critical opportunities to address SDH.

### Theme 1: Financial Insecurity and Inadequate Access to Resources for Everyday Social Needs Negatively Contribute to Caregiver and Child Health and Well-Being

Caregivers consistently identified financial resources as a significant unmet social need that caused stress and impacted both caregiver and infant health and well-being. Financial insecurity compromised access to social needs that included transportation, stable housing, childcare, clothing, food, hygiene products, safe sleep, and safe living spaces. Caregivers who had lower income (<30k per year) described the emotional toll and mental burden that lack of access to social needs further contributed to the underlying stress of having a preterm infant, as well as various strategies they used to manage and address financial strains. Caregivers described their attempts to distribute limited resources across their family, and still, social needs persisted. Further, caregivers described the burden of attempting to address unmet needs and the accompanying experience of isolation. As 1 caregiver described:

“Navigating the homeless system, which is extremely hard. The resources are definitely lacking there, and the support is definitely lacking there. I had to do a lot of legwork, even with having some support. I did a lot of work by myself which made things more challenging because it shouldn’t be all on the people that are struggling to have to do all the work.”

- C8

Levels of social support varied substantially among caregivers and served to protect against or exacerbate financial related stressors. One caregiver described isolation and lack of support systems only amplified financial insecurity issues. For her, the consequences of unmet social needs meant adverse outcomes for both her and her infant. Here she described unmet needs interfering with her engagement in desired health behaviors like breastfeeding:

“I remember crying the first time I had to wash my daughter’s clothes because I’m thinking she’s a preemie, her immune system, she’s susceptible to all these things. I don’t want her to be in the public. But now we got stains and poop on all our clothes, and they need to get washed. I don’t have a home right now. I have to go to the public wash house with my preemie daughter. I literally was in tears because of that… It just made me realize I’m completely alone…Being in stable housing… is most important. When you have babies and are looking for a place to sleep, you cannot breastfeed your baby. This was the hardest for me.”

- C12

A few caregivers also described lack of transportation as a major obstacle for engaging in health care appointments which subjected herself and her child to unsafe spaces. On the contrary, caregivers who had more financial means (>100k income per year) and social and financial support from family members described the benefit of additional diagnostic evaluations and therapies through private schools and companies for their children and expressly acknowledged the unjust nature of inequities limiting availability for all children:

“He’s in a private school. We’ve paid to have a neuro-psych evaluation done. He has a therapist. He’s got a coach. He’s got the therapist at school. Like, he’s super well-resourced, right now, right, because we can afford to do that. And, you know, arguably, because we can afford to do that, even if we didn’t do it, he was going to be okay, right? It’s not fair that, because I have money, or I have a health insurance plan that covers it, my son gets to have all these things, and just because the luck of the draw, you don’t have those resources, you don’t get that. It’s hard, right?”

- C4

### Theme 2: A Trusted Provider Who Takes a Personal Approach to Screening and Addressing SDH is Needed in Medical Settings

There was a noteworthy variation in whether caregivers had been screened for SDH in the NICU and primary care settings and how they were screened and approached. Caregivers had many experiences to reflect on due to experiencing several transitions of care, including transfers between local hospitals and inpatient to outpatient care across several medical settings. Caregivers generally appreciated being screened for SDH as they recognized the potential benefit and intervention. The most frequent recommendation was in-person screening by a trusted medical staff member with whom they have a regular consistent relationship of communication, that is, separate from the medical team, like a social worker:

“You have a regular, consistent relationship of communication, and they’re not, quote, unquote, doing the care. So there’s not that white coat syndrome barrier. But they feel more like a collaborator or a creative thinker in the way that they approach supporting us, the families, and the kids. But I think that works because they have this long-standing relationship, and you’re going to see them every couple of days or on a regular schedule. And they’re not diagnosing your baby or treating you.”

-C3

Caregivers also recommended that physicians and nurses should have increased awareness of what SDH resources exist and how caregivers can access them, so they can be a secondary information source to social workers. Several best practices were identified for screening, including a personable approach that leads to establishing rapport, respectful conversation without judgment, and a stated goal to support. Without a personal connection before discussing sensitive subjects, the staff risk offensively screening and providing resources by passing judgment, blame, or displaying superiority. In addition, caregivers were aware of the potential risks of disclosing social needs, such as child protective service involvement and separation; therefore, establishing safety is paramount. Although the approach was important, concrete and tangible resources were also equally desired:

“Don’t just empathize with me, actually help me out here.”

-C19

Examples of timely access to readily accessible resources on hand include pamphlets with resources and contacts previously vetted to be available and reliable:

“I think having resources on hand…Having something like—that’s what I plan on doing—having a pamphlet where it shows, okay, for housing, these are the resources and the contacts. But having resources that someone had already contacted so they’re reliable resources.”

-C1

A few caregivers described pride or guilt as obstacles to obtaining resources, knowing other caregivers may need resources and feeling uncertain if resources are finite. They described a potential solution and benefit to education and reassurance to be provided during resource screening and provision. Caregivers preferred money for autonomous determination and flexibility of how it would be best to support their family.

### Theme 3: Inequitably Distributed, Fragmented, and Disorganized Medical and Social Support Systems in the Transition to Home Period is Burdensome and a Source of Stress

Caregivers describe high administrative and cognitive burden associated with coordinating medical and social care during the transition to assuming the responsibility of primary caregivers after their infant’s discharge from the hospital. Organizing and attending many medical appointments and therapies was common and challenging. Caregivers reported attending multiple weekly medical and therapy appointments across different locations, which absorbed their limited time and resources. One caregiver described the abrupt severing of resources at discharge after being inpatient, in her case, her mental health support,

“Right when you walk out the hospital, y’all relationship is over. So like, you feel me? So like I told you, he spent … 62 days in the hospital, and I had all those resources for those 62 days, but the moment I walked out those doors, like, everything stayed at the hospital. You know what I mean? So like, I went home with the same PTSD that I was getting, like, you know, help for because she had a therapist in the hospital that would talk to me and stuff like that.”

-C5

In tandem with navigating complex dyadic medical and social care, resources were inequitably distributed through these fragmented and disorganized medical and social support systems. Caregivers described hearing about resources from their peers that were not offered to them and the barriers to accessing those resources. Barriers included differing offerings and awareness of existing resources and differential accessibility across counties, medical systems, and insurance coverage. In 1 instance, a caregiver described a:

“Public health nurse, they would come out for a lot of the other girls that were in the shelter. But I could never get one. They were backed up or they didn’t have any availability … so I never could get one. But I felt like that would have been something nice to have”.

- C12

In models that worked well, caregivers described wraparound services received in primary care settings dedicated for children who have been hospitalized in the NICU. One caregiver described receiving this type of care at their pediatrics office:

“They don’t let us be in any kind of need. Anything that we need, they give me a social case manager so I can ask for anything that we need.”

-C11

When imagining an ideal support system, they described transportation resources and optimized and coordinated appointments that must be in-person and virtual appointments when appropriate. They additionally named continued lactation and mental health services, donor breast milk and hospital-grade pumps, check-ins with home nursing, doula, peer support, or other personnel support for at least 6 months to assist with recovery of caregiver and infant.

### Theme 4: Community-Based Organizations Centering Black Families Holistically Address SDH and Promote Social Well-Being and Connectedness

Caregivers enthusiastically participated in several Black-centering community-based organizations (CBOs) of private, regional, and state varieties. Specific CBOs that were mentioned included Black Centering, Black Infant Health, Perinatal Equity Initiative, Melanated Mama Meals, Black Women Birthing Justice, Abundant Birth, Rafiki Coalition, Embrace, Ujima Adult and Family Services, Preterm Birth Initiative, and CoCo Doulas.^26–36^ These organizations serve a variety of purposes across prenatal to postnatal settings but generally support Black caregivers and their infants before and after birth. Caregivers reported that these organizations provided services that met a range of their social needs including gift cards, air filters, strollers, car seats, diapers, home-cooked meals, doulas, and childcare. Several of these organizations are Black-owned or led by Black community leaders and have racially concordant teams providing services. One caregiver described peer support services in Black Infant Health:

“We’ll talk about, like, you know, how Black mothers are treated in the healthcare system or, you know, just, like, statistics that, you know, affect us or whatever, and so, like, that was informative. And then the lady was a go-getter ‘cause she was, like, dropping off diapers and diaper bags. And whatever we needed, she had it. Like, you know, and even for my older kids, she’s like, ‘If you need a mattress, I can get you a mattress’ … I really didn’t need for nothing before or even after because, like, they were there on top of… my supportive family.”

-C5

A caregiver’s isolation was addressed by Beloved Birth Black Centering prenatal care:

“It was so helpful. You didn’t feel so alone. You got to meet other Black mothers who were going through the same thing that you were going through and around the same—you know, they grouped us according to how far along we were.”

Other programs that were not specifically Black-centering also provided social support:

“Now, we are still working with Catholic Charities. For instance, they help us with utilities. They help us with rent. They help us with clothes, childcare. I think they have a lot of resources like Compass Family, Homeless Prenatal Program. I think it’s fantastic. They really helped me and they gave me all the resources that I could ask for at that time”.

-C11

### Theme 5: State and Federal Government Legislation, Policies, and Programs Are Critical Opportunities to Support SDH

Caregivers offered many examples and opportunities for state and legislative bodies to support previously identified social needs. One commonly identified policy was parental leave:

“I think there should be more parental leave, like six months minimum for men and women, just parents. Period. Because the people who are supporting you, they need help, too. It’s a lot for them to take on too. I need that. I think that needs to happen. And I wish there was more funding for at-home visits right after the baby is born because I had a few of those.”

-C3

Another caregiver identified the unrealized opportunity for policies to specifically support caregivers with preterm infants and infants with long NICU stays. Families described parental leave in other countries as evidence of feasibility and the potential benefits of parental leave:

“In Canada, [they] basically give you your income for practically a year, and your job is protected for you to be gone for a year and then come back to your job. I mean that’s tremendous. I mean people don’t have that here, and it really forces them to make hard choices. And some of those are like, I’m going to stay home but it’s at great financial cost. And maybe I’m going into debt. And all of those things, I think, are really taxing on the parents. And whatever taxes the parents has the potential to be compromising for the child… Society needs people to have kids, and yet they put the total burden for it on the people.”

- C14

The importance of parental leave to impact bonding was described by 1 caregiver:

“And babies need an opportunity to have a chance in this world, and the way they do that is really bonding with the people who are their first family.”

-C3

Other opportunities for state and federal governments to support families included economic relief policies specific for caregivers with hospitalized or preterm children for social needs such as gas, food, parking, childcare, and mental health support. Basic guaranteed income was highlighted as a federal policy addressing these social needs, allowing caregivers to determine where funds would be best invested. Caregivers appreciated several existing state and federal services like public health nursing, shelter services, Section 8 housing, Special Supplemental Nutrition Program for Woman, Infants, and Children, Medi-Cal, parks, and green spaces. However, some caregivers experienced gaps in awareness and exposure, access, and connection gaps with these services. Similarly, mandated developmental support through regional centers is available to Californians, but caregivers experienced long wait times, coordination challenges, and disqualification after their child reached age 3.

## Discussion

In this study, Black caregivers with preterm infants described their experiences with gaps, opportunities, and successes for screening and addressing SDH across socioecological levels with racially concordant researchers. At the individual level, financial insecurity and social needs remain substantial barriers to thriving long-term after preterm birth. At the interpersonal level, when screened in medical settings, caregivers preferred a trusted medical provider who takes a personal approach with readily available resources. At the institutional level, inequitable distribution, fragmentation, and disorganization in these resource-constrained systems contribute to gaps in resource provision needed for families to thrive and leave opportunities for substantial improvement. At the community and policy levels, CBOs that center Black families, state and federal governments, nonprofit organizations, and medical systems all provide critical support to Black families with preterm infants and have opportunities to grow and expand their reach and impact.

Our study highlighted the experience and impact of ongoing unmet social needs of Black caregivers who had preterm infants and spent time in the NICU. A few recent studies have described this hardship quantitatively, and research on the SDH in the NICU is generally new.^11,37–39^ Our study adds to previous literature by providing additional rich detail of experiences and priorities from the perspective of Black caregivers who are racially marginalized and disproportionately impacted by social and medical complexities long after hospital discharge. Other qualitative studies interviewing Black caregivers with preterm infants have tended to focus on the hospitalization period^16^ or on different specific topics like racism,^17–19^ stress,^20^ and/or mental health.^21^

Screening and intervention for unmet social needs is essential for adequately addressing racial health care disparities in the NICU setting. There is no uniform national standardized screening tool or strategy for screening in pediatric or neonatal populations, but the literature around best practices is evolving.^40^ Although standards-setting, regulatory, and quality initiatives have been launched that require health care systems to screen for SDH, knowing the priorities of the community being served is recommended before implementation.^41,42^ Our study adds Black preterm infant caregivers voices to that conversation. Our study aligned with previously identified barriers to effective screening and provision of resources, including justified fears of repercussions after disclosing social needs and guilt of utilizing limited resources ^43,44^ but also added other insights to barriers such as caregiver pride. Literature regarding preferences for the screening methodology (ie, electronic or paper forms, in-person) are mixed. For example, one recent study highlighted family preferences for electronic screening as a preferred modality but in-person screening was also acceptable to many caregivers.^45^ A randomized control trial also found that electronic screening had marginally higher disclosure compared with face-to-face interviews and suggested an electronic method to report sensitive information without judgment may be more effective.^46^ Other caregivers, including our study participants, preferred humanistic in-person screening by a trusted social worker.^45^ Generally, trust is an important factor for perceived appropriateness of screening of caregivers in a medical setting.^47,48^ Clinical stakeholders also have noted that there are more frequent opportunities to build relationships and trust and to screen for SDH during a hospitalization period, like in the NICU.^49^ Importantly, prior experiences of discrimination that impact racially marginalized communities can impact levels of trust in the medical system and SDH screening and interventions can be opportunities to build trust.^50^

Black-centered CBOs are uniquely prevalent in this area, specifically California and the Bay Area. Although several Black-centered CBOs have materialized, data supporting their efficacy are needed. Preliminary survey data evaluating a CBO mentioned by participants, Black Infant Health, are supportive of improving SDH-associated outcomes such as empowerment, social and emotional support, stress management, healthy behavioral modifications, food insecurity, and depressive symptoms.^51^ Our study adds to limited published data evaluating these novel services, describing caregiver enthusiasm about the gaps Black-centered CBOs have filled to meet their SDH and emotional needs.

Caregivers experience financial burden, financial worry, and related employment decisions after preterm birth and NICU discharge.^52,53^ State and federal legislative policies such as paid family leave and economic relief such as guaranteed basic income or unconditional cash transfers have been advocated for by scholars and show promise for improving birth outcomes and promoting infant health postnatally.^54–59^ Paid leave has been associated with improved breastfeeding rates, parental mental health, infant health, and mortality.^60–63^ Preterm birth is negatively associated with return to work and paid leave has been proposed to improve outcomes in this population.^53,59^ However, there are sparse studies on the impact of paid leave after preterm birth and there are unique challenges in studying the impact of perinatal legislative policies on health outcomes.^53,62,64^ During pregnancy, Black families in the Bay Area can be financially supported by guaranteed income through the Abundant Birth Project; evaluation forthcoming.^32^ This project is a guaranteed income program that provides monthly income supplements based on the county of residence during pregnancy for those at highest risk of preterm birth in California, to support healthy pregnancies.^32^ Randomized control trials utilizing unconditional cash transfers for caregivers utilize socioeconomic status or prematurity alone as their criteria for eligibility in Chicago and Boston with the purpose of improving breast milk provision and skin-to-skin care, known critically important factors for infant health and development.^55,65^ These early studies will provide insight on short-term financial perinatal support, and there remains opportunity to study extended financial support beyond the NICU to support the long-term health of NICU graduates. Medi-Cal insurance coverage, highlighted by participants, and expansion efforts could improve access to care and benefits for underserved populations.^66^

Our study participants validate existing and prevalent observations regarding disorganized and fragmented care and coordination in the US health care– and US government–funded systems that are not new.^67^ In the Bay Area, perinatal services are available, but the dispersed and uncoordinated nature of services makes it hard for the full range of community members to access them.^68^ Experiences with racism and discrimination while accessing these services also limit their reach and efficacy.^68^ Hospital systems and payers should have interest in addressing SDH and improved care coordination in high-risk groups; as lower socioeconomic status is associated with higher health care utilization.^39^

### Limitations

Our study captured the perspectives of socioeconomically diverse Black preterm infant caregivers who had experiences across several hospital systems and counties in the Bay Area. Extrapolation to a specific hospital system or beyond the study’s demographic, geographic, and political landscapes is therefore limited. We found wide variations across twenty caregiver’s experiences and within the themes. Although we found saturation in the themes, the variation may be better understood in a larger sample size. Recall bias was possible given the long eligibility period, although the vivid descriptions of their experiences suggest that they were memorable. Social desirability bias and power dynamics were potential sources of bias, predisposing caregivers to either over- or undershare personal experiences. The interviewers were trainees and shared racial and often gender identities with interviewees lessening the risk of power dynamics. This study may be subject to selection bias, as caregivers who faced notable barriers to SDH may be more likely to participate. Our study required video recording and thus may have excluded families without this technology access.

In conclusion, SDH are a significant source of stress for Black caregivers after preterm birth, and there are ample opportunities across state and federal legislative policies and programs, CBOs, medical systems, and connections across the systems to address them. Future studies are needed on the impact of screening and addressing the gaps identified in short-, medium-, and long-term outcomes on this community.

## Supplementary Material

Supplementary Material

## Figures and Tables

**Table I.** Sample characteristics

Demographics at time of study	Subcategories	n	%	Mean (range)
Caregiver relationship to child				
	Mother	18	90	
	Father	2	10	
Caregiver age	25–34	10	50	
	35–44	6	30	
	≥45	4	20	
Caregiver gender	Female	18	90	
	Male	2	10	
Caregiver race	African American/Black	15	75	
	Black African	2	10	
	Afro Latinx	1	5	
	African American/Native	1	5	
	Afro Caribbean	1	5	
Caregiver nativity	Born in the US	18	90	
	Born outside the US	2	10	
Caregiver first language	English	19	95	
	French	1	5	
Caregiver income per year	<10k	6	30	
	10k-49k	4	20	
	50k-100k	4	20	
	>100k	5	25	
Caregiver education	High school	3	15	
	Some college	5	25	
	College graduate	4	20	
	Professional degree	7	35	
Caregiver employment	Full-time	7	35	
	Part-time	2	10	
	Unemployed	7	35	
Caregiver insurance	Public	10	50	
	Private	9	45	
	None	1	5	
Child gestational age in weeks at birth: (mean [range])				27.5 [23–35]
	23–25	6	30	
	25–28	4	20	
	29–32	6	30	
	32–35	4	20	
Child age in years				6 [0–12]
Birth hospital	Alta Bates	8	40	
	Kaiser Oakland	1	5	
	Alameda County Medical Center	1	5	
	Kaiser Santa Clara	1	5	
	Marin General	1	5	
	Saint Francis Hospital (Dignity Health)	1	5	
	Kaiser (no specification)	3	15	
	UCSF Medical Center	4	20	
Total		20	100	

**Table II.** Themes and exemplary quotations

Themes	Exemplary Quotations
Individual socioecological level: Financial insecurity and inadequate access to resources for everyday social needs negatively contribute to caregiver and child health and wellbeing.	“Actually, when my wife was pregnant, we couldn’t, let’s say, take care of the stove, like feeding and the rest, the education, the bills. So an important factor now is financial stability now. As the head, as the father, it’s my responsibility to take care of both my wife and child. So when I’m unable to do this, it affects my wife and my child. So I needed to work hard, so my baby and my wife can feel fine, feel comfortable. The father’s well-being is important, it’s critical.” As a family, we try to combine everything, but she can’t use everything I can use. And she can’t eat everything I can eat. She can’t wear everything I can wear. She can’t use the same detergent as me. So it’s like she has to have her own set of necessities, and that’s like a whole other thing.” “You need a warm place. You need a warm place to be able to think about what you are going to do. But, while you are on the street, you cannot think about anything.” “Transportation was a huge issue. I was in between places. I don’t have a car. I’m not a driver. So that was really stressful, wondering how I’m going to get to my appointment. If it’s at 8:00 AM in the morning, and the bus doesn’t run until 9:30, or the train doesn’t run, whatever. And then it’s super dark out and cold and stuff. Early in the morning, you can barely see. Like, I remember having to take her to follow-up appointments super early. The shelter was in the Bay view. And that’s such a sketchy neighborhood. And just being up where it’s dark and you’re scared. And I think that was super stressful. And I’m sure my baby is still dealing with that because I was always stressed. Transportation was hard.”
Interpersonal socioecological level: A trusted provider who takes a personal approach to screening and addressing SDH is needed in medical settings.	“In general, especially when it comes to asking about those needs for support with social drivers of health, just first getting to know you, establishing some rapport, entering with respect, and being personable, and first getting to know you as a person, and then explaining why they’re asking those questions with the goal of just trying to support you and to support your baby and your pregnancy.” “So I was saying that they never asked me in a genuine kind of way, about that, saying, hey, before we start your care, let me ask you a couple of questions, so I could better care for you. It was just made to sound derogatory or they’re insinuating things about me. But they never asked me those things to try to get to know me or know how they could help me. It’s just like, oh, I don’t see Dad. Like, where’s Dad? Instead of just being like, hey, do you have some support? What neighborhood are you from? What resources are there? What’s your eating been like since you’ve been pregnant? How are you feeling? That sort of thing. No, it was just always something negatives, or to blame me. But it was just never communicated in a respectful way, just to say, I want to know more about you so I could take better care of you.” “I think that… I don’t know. I think that’s hard. I think when you talk about 2 social workers or one and a half for an entire NICU of families, whether it’s full or not, it’s not that much capacity. So it feels like, oh, what’s the capacity here? But also, just what are their levers for being supportive and helping to make change?”
Institutional socioecological level: Inequitably distributed, fragmented, and disorganized medical and social support systems in the transition to home period is burdensome and a source of stress.	“No public health nurse was assigned to me, even though I had a premature baby and I’m living in a shelter. All those things I feel like played a role in why my daughter wasn’t able to do certain things when she should have been able to, or while I was so stressed, trying to figure out, well, how come you’re not talking, how come you’re not doing this or that? […] I had to Google things and buy books. And I had to pay for online classes to try to learn how to teach my baby certain stuff. And I just feel like that’s absurd. If I have health coverage, and that’s what they’re there to help with, and I’ve seen them help so many other moms with that.” “And then they kind of just don’t check in. And I know NICU’s busy, but it would be like, you guys don’t care anymore. You watched my baby for 3 months or a year or however long. Lots of people were there for a long time, and nobody kind of really checks in after that. You’re kind of just on your own. And I don’t think that we’re really fully equipped sometimes for that transition for how it is at home. Nobody checks in. It’s extremely, extremely challenging. I think I did well for the first couple of months of her being home, and then I got extremely depressed because it was a lot, and I think the constant worrying, and I mean, because you already worry as a parent anyway, but just it’s intensified when you have a baby that was born premature and stuff.”
Community socioecological level: CBOs centering Black families holistically address social drivers of health and promote social well-being and connectedness.	“The Black Health Centering Group was amazing because there are certain things within African tradition that your parents just don’t talk about.” Re: Black Infant Health: “I ended up getting connected to Black Infant Health in Contra Costa, which is just an amazing organization. They connected me to a lot of resources, and so I’m so grateful, one of them being childcare because that is like the hardest. Besides keeping a roof over your head, it’s just ridiculous how much childcare is, how much it costs, and how expensive it is. And so just having someone to explain certain things and explain how things work to me, it just really made a big difference in the impact.” “So one big one that I use that I got in touch with was Melanated Mama’s Club, and they are very helpful just with meal preps. They send meal preps to you, they send you guides on how to eat better as far as having a nutritionist. You’re able to speak to a doula there for free and have sessions with her just to kind of educate you on different things, even if you’ve already had your baby because in my situation, my baby was already here. And just educated me on different types of herbs, different things to eat to build up production of milk in your body. Just a lot of different resources as far as sending you different foods that are actually tasty and that you enjoy, different vegetables and fruits that are in season, and sending you a log that shows you different meals you can make with the things that they already sent you.”
Policy socioecological level: State and federal legislation, policies, and programs are critical opportunities to address SDH.	“I think more leave before because you had to choose between taking the leave before or after, and when you have a baby that’s going to be born so early and be in the NICU so long, you need more time. So it would have been nice to have more medical leave before the baby was born, and that was paid for.” “I think that, in general, the lack of laws that support pregnant people and parents. You know, they impede my child’s wellbeing and health and every other child’s. You know, the fact that I was in the midst of a high-risk pregnancy and there weren’t social supports that said, ‘You could just not work while you’re pregnant,’ and especially if it’s high-risk. I felt like I had to work, and I had a very demanding job at that time. And maybe it would’ve been better to just not work or work less and prepare and take care of oneself. And after giving birth, I didn’t go back to work. But this was a privilege. It wasn’t something that was supported by policy.” “They’re very, very sick in the first 2 years, and there’s no, like, no family medical leave. Like, they don’t even count, like, a preemie as like you need time off. So that was very hard. Like, I had to pretty much, like, go without pay because he was, like, in and out of the hospital. So when you’re in and out of the hospital with your NICU, you don’t get any income after that. My doctor was able to, like, because I had health issues, she was able to extend my medical leave to cover me. But there wasn’t anything to, like, for my son. So when I got exhausted, I couldn’t go back and say, okay, I need this, you know, because my son’s in and out, or my daughter’s in and out of the hospital. That’s something that needs to—because it’s those, like, those policy things that make it harder for you, like, because there are a lot of issues. There’s a lot.” “[There’s] no public policies in place that caters to women who have preterm births. So like everything is just a standard. You get the 6 weeks for yourself to recover and then depending on how you deliver the baby. But I think that there should definitely be something carved…mothers who do have preterm babies because the NICU stay can differ depending on how early the baby is, how sick the baby is, and all those other factors. And there’s nothing to kind of protect us to continue to have that financial support during that time.”

## Abbreviations

CBO: Community-based organization
NICU: Neonatal intensive care unit
SDH: Social drivers of health
