# Supplementary material for: Gaps, Successes, and Opportunities Related to Social Drivers of Health from the Perspectives of Black Preterm Infant Caregivers: A Qualitative Study
Source: J Pediatr. Author manuscript; Available in PMC 2025 Jul 7. (PMC12233135; doi:10.1016/j.jpeds.2025.114598)
Supplement: Supplementary Material [file NIHMS2084780-supplement-Supplementary_Material.docx]

**
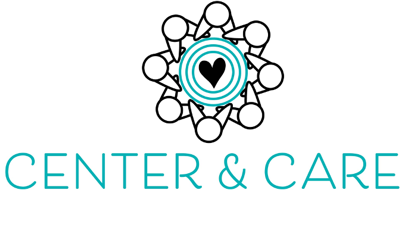
**

Centering Black Preterm Infant Caregiver Priorities 
**CENTER & CARE Study** 
**Interview Guide Script**

My name is ___, I work with the CENTER & CARE research team at UCSF. We are carrying out a study to identify family priorities to support the health and development of Black children who were born preterm. Your input is valuable to help us understand how our society and healthcare systems can better support families after their children are born preterm.

*GO THROUGH CONSENT AND ADMINISTER SHORT STRUCTURED QUESTIONNAIRE FOR DEMOGRAPHIC DATA IF THEY DID NOT ALREADY CONSENT AND PARTICIPATE IN SURVEY.*

*TURN ON RECORDER.*

My name is ___ I am with respondent #

| Main Questions: | Specific Probes | General probes |
| --- | --- | --- |
| 1. Tell me a little bit about yourself and your child. | - How old is your child now?  - How long did your child spend in the NICU? |  |
| 2. Tell me about how your child is doing today? | a. How is their health?  b. What activities do they do? | a. Can you tell me more about that?  b. What actually happened?  c. Can you walk me through what happened, step by step?  d. Can you give me an example?  e. What makes you say that?  f. How did that make you feel? |
| 3. How would you describe “thriving” or the best possible life for your child? |  |  |
| 4. a. What are the most important factors to impact to your child’s ability to thrive before they were born?  b. What are the most important factors to impact to your child’s ability to thrive after they were born?  c. What **interpersonal factors,** such as family, friends, social networks were/are important for your child’s long-term health?  d. What **organizational** factors, such as schools, workplaces, church are important for your child’s long-term health?  e. What **community spaces/environmental factors** were/are important for your child’s long-term health, such as air they breathe, water they drink, access to playgrounds?  f. What **public policies,** such as state/national laws are important for your child’s long-term health? | Did any of these factors change for you from before baby was born to after baby was born? |  |
| 5. What do you feel like you needed, but didn’t/don’t have, to help your child thrive before & after they were born? |  |  |
| 6. We know that parents and children’s health are linked. What other factors do you think are necessary for parents/caregivers to thrive after preterm birth? |  |  |
| 7. Thinking back to the time surrounding your baby’s birth, what feedback would you give to the medical community to support you and your family? | Consider having interviewee specify what period surrounding baby’s birth (prenatal visits? While laboring? Shortly after baby was born? Longitudinally through NICU stay?) |  |
| In the next few questions I will be asking you about social drivers of health, which is a term many people are not familiar with, so I will define it here:  Social drivers of health (SDH) are conditions that impact a person’s health, like their financial means, the physical environment they are in, and the social connections they have. Examples of SDH include: income, employment, housing, access to high school or college education, access to healthy foods, exposure to crime/violence or racism/discrimination, and access to healthcare. | If families need more examples or a list, please provide them with the SDH table. |  |
| 8. Were you asked about social drivers of health in your child’s primary care clinic (the medical office where your child received medical care)? | **If yes,** ask the following questions:   1. What was that experience like for you? 2. If positive, what part of it made it a positive experience? 3. If negative, how would you improve that experience? 4. Did the clinic help you locate resources to address a social driver of health? Which drivers, and what type of resources were given (for each driver)?   Did you feel resources were provided in a timely manner?  Was there any follow-up after resources were located/provided.  What team members, if you can recall, were helpful in providing resources (social worker, case manager, medical team members, etc).  What factors would facilitated comfort about being asked about SDH & providing resources?/ In what ways would it be helpful to be asked about SDH? (in-person, online, resources given first)  **If no,** ask the following questions:   1. Do you wish that you were asked these questions? Why or why not? |  |
| 9. Were you asked about social drivers of health while your child was in the NICU? | **If yes,** ask the following questions:   1. What was that experience like for you? 2. If positive, what part of it made it a positive experience? 3. If negative, how would you improve that experience? 4. Did the NICU help you locate resources to address a social driver of health? Which drivers, and what type of resources were given (for each driver)?   At what points during the NICU stay were social drivers of health addressed? (Such as perinatally vs throughout NICU stay vs prior to discharge, etc).  Did you feel resources were provided in a timely manner?  Was there any follow-up after resources were located/provided.  What team members, if you can recall, were helpful in providing resources (social worker, case manager, medical team members, etc).  **If no,** ask the following questions:   1. Do you wish that you were asked these questions? Why or why not? |  |
| 10. What would make asking addressing social drivers a more helpful or comfortable experience in the healthcare setting? |  |  |
| 11.What would be better to improve with transition to home care?  12. Is there anything else that we have not discussed that you think would be important to know regarding your baby’s experience? | 1. SDH, general support |  |

This is the end of our interview. Thank you so much for taking the time to answer our questions.

Do you have any questions for me about this interview?

What was it like for you to participate in this interview?

It was a pleasure to speak with you today.

Please contact us if you have any questions or concerns: [centerandcare@ucsf.edu](mailto:centerandcare@ucsf.edu)

Summary Impressions and Comments (for interviewer or research team only):
